# Color agreement between try-in paste and resin cement: Effect of thickness and regions of ultra-translucent multilayered zirconia veneers

**DOI:** 10.15171/joddd.2019.010

**Published:** 2019-04-24

**Authors:** Mehdi Daneshpooy, Fatemeh Pournaghi Azar, Parnian Alizade Oskoee, Mahmoud Bahari, Saeede Asdagh, Seyed Reza Khosravani

**Affiliations:** ^1^Department of Operative Dentistry, Faculty of Dentistry, Tabriz University of Medical Sciences, Tabriz, Iran; ^2^Dental and Periodontal Research Center, Department of Operative Dentistry, Faculty of Dentistry, Tabriz university of Medical Sciences, Tabriz, Iran

**Keywords:** Color agreement, resin cement, try-in paste, ultra-translucent zirconia, veneer

## Abstract

***Background***. The current study aimed at identifying the color agreement between try-in pastes and the respective resin cements and investigated the effect of thickness and regions of Ultra-Translucent Multilayered Zirconia Veneers.

***Methods***. A total of 90 cubic zirconia discs were prepared at two different thicknesses (0.5 mm and 0.7 mm) (n=45) in five groups in terms of the shade of the try-in paste and resin cement as follows: Universal, Clear, Brown, White and Opaque. Try-in paste and the respective resin cement were applied between the specimens and composite substrate, respectively, and colorimetric evaluation was carried out using CIE-Lab system. For each specimen, ΔE between the try-in paste and cement was calculated. Data were analyzed with SPSS 17 using Multifactor ANOVA (P<0.05).

***Results***. Multifactor ANOVA results showed that ΔE values were significantly affected by the resin cement shade and the thickness of ceramic veneer (P<0.05). The results showed better shade agreement between the try-in paste and the respective resin cement with thicker ceramic veneers. The results of Tukey HSD revealed that ΔE values for the Clear, Universal and Brown shades were less than those of the White and Opaque shades. Lighter shades exhibited better agreement between the try-in paste and the respective resin cement.

***Conclusion***. Perceptible color difference was found between the try-in pastes and the respective resin cement in most colors investigated. Although, the agreement of the try-in pastes and the respective resin cement was affected by the thickness of zirconia veneers, the different regions of multilayered ultra-translucent zirconia ceramic showed no significant effect.

## Introduction

The esthetic expectations of patients for achieving good results in anterior teeth necessitates materials with optical properties similar to natural teeth.^1^ At present, there is a sustained effort by dental practitioners and ceramists to obtain superior results, especially with ceramic veneers.^2^ Due to fast improvements in both biomaterials and processing technologies, more esthetic solutions are possible.^3^ Versions of “high-translucent” or “cubic-containing” zirconia are commercially supplied recently, produced with major modifications in microstructure and composition to increase their translucency without significantly losing their fracture resistance, thereby expanding their clinical indications, including veneers and ultra-thin veneers.^4^ Currently, multilayered translucent zirconia which is internally colorized has made it possible to produce monolithic veneers with optical properties to precisely resemble natural tooth without applying any porcelain layer.,^6^ Ultra translucent multilayered zirconia (UTML) is designed to have a minimal thickness of 0.4 mm for veneer.^3^

One other important factor in esthetic rehabilitation with all-ceramic restorations is the choice of resin cement, as it can affect the color of the final restoration.^7^ According to some studies, the resin cement affects approximately 10‒15% of the optical results of all-ceramic restorations.^1^ To obtain better predictability of esthetic results, veneer try-in should be performed prior to cementation. The dentist and patient can evaluate color changes in the tooth/veneer system using try-in pastes that correspond to the same shades of resin cements; moreover, the esthetic expectation is also achieved.^8^

The shade agreement between the try-in systems and respective cements has been evaluated in different studies, but no significant differences have been found.^8-12^ Xing et al reported that the shade of try-in paste and the corresponding resin cement attained high agreement. They suggested that the application of try-in pastes could practically predict the resin cements’ final esthetic result in veneers.^11^ On the other hand, some studies found no color matching of resin cements and corresponding try-in pastes.^13-16^ Mourouzis et al^15^ indicated that dentists should not count on try-in pastes for the final color evaluation^.^ Therefore, knowing whether try-in pastes are reliable regarding the final color of a restoration is a factor of greatest importance in an esthetic laminate veneer treatment.^8^

Different ceramic systems can have different effects on try-in paste and resin cement agreement. Vaz et al^12^ showed no color differences between the try-in paste and resin cement in leucite-reinforced glass ceramic (Empress). Xu et al^14^ reported no similarity of colors between the try-in paste and resin cement in lithium disilicate glass ceramic specimens (Emax). Although Rigoni et al^13^ showed no color agreement between try-in paste and resin cement in nanofluoraptite ceramic discs, Xing et al^11^ reported excellent try-in paste‒resin cement agreement in Ceromer discs.

Since few studies on zirconia veneers have been reported,^17^ and inadequate data is available about multilayered ultra-translucent zirconia and its possible effect on try in paste–resin cement color agreement, the current study was carried out to evaluate the agreement of shade between try-in pastes and resin cement for two thicknesses (0.5 and 0.7 mm) in different regions of multilayered ultra-translucent zirconia. The null hypotheses of this study were: 1) There is no perceptible color difference between the resin cement and corresponding try-in paste) 2,3) The thicknesses and different regions of multilayered ceramic veneer have no significant effect on the agreement of try-in pastes and the respective resin cements.

## Methods

### Preparation of Ceramic Specimen

**Figure 1:**
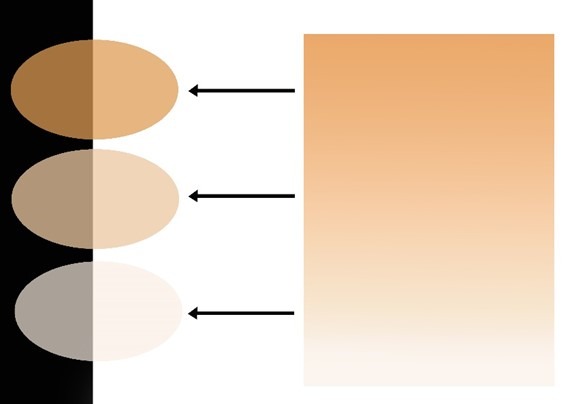
Schematic representation of the multilayerd disc.

The pre-sintered cubic zirconia material selected for this study was Katana, A1, T14, UTML (Ultra Translucent Multilayered) shade A1 (KATANA^TM^ Kuraray Noritake Dental Inc., Miyoshi, Japan). A total of 90 thin slices were provided in two thicknesses for each 45 samples (a: 8×11×0.5 mm; b: 8×11×0.7 mm) using CAD/CAM milling (pixdent, Bonyan Mechatron, Iran), and sintered based on the manufacturer’s recommendations, and all the specimens were polished with 1200-grit silicon carbide paper(Figure 1).^8^

### Substrate Fabrication

Ninety resin-based composite specimens (A3 shade, Natural Shade, NOVA DFL, Rio de Janerio, RJ, Brazil) were fabricated using a rubber mold (8×11×5 mm) to obtain a uniform background and used as the background color to mimic the prepared tooth substrate.^18^

### Try-in Paste Application

**Figure 2:**
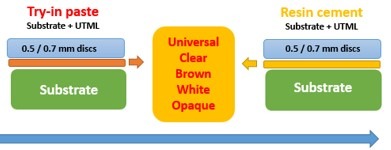
Flowchart of the experiment design.

In each group (0.5, 0.7 mm) the specimens were divided into five subgroups according to the shade of the try-in paste and resin cement as follow: Universal, Clear, Brown, White and Opaque (Panavia V5 Kuraray American Inc.). The try-in paste was applied between the ceramic veneer and composite substrate. To obtain uniform film thickness of try-in paste, a pressure of 250 g^19^ was loaded over the ceramic veneer using a universal testing machine (Hounsfield 5K, England). Colorimetric evaluation was carried out with an Spectroshade spectrophotometer (SpectroShade Micro, MHT, Verona, Italy). Then, the try-in pastes were removed from the specimens with a flat-angled brush (Figure 2).^8^

### Cementation of Ceramic Discs

**Figure 3:**
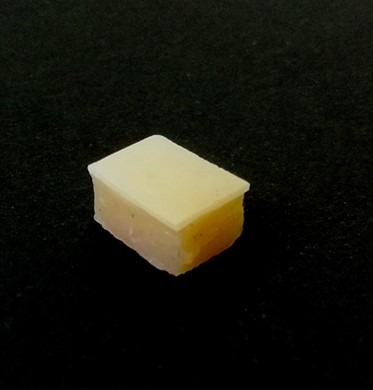
UTML cemented on composite resin substrate.

Before cementation, the zirconia specimens were abraded with airborne particle for 10 seconds with 50-mm alumina at 0.2 MPa. Then, the specimens were water-rinsed and air-dried. A primer containing silane and MDP (Clearfil Ceramic Primer plus; Kuraray Noritake Dental) was applied to all the pretreated ceramic surfaces for 20 seconds, followed by air thinning for 10 seconds. A tooth primer (Kuraray Noritake Dental) was applied to substrate surfaces for 20 seconds, followed by air thinning for 10 seconds. Then, five different shades of resin cement (Panavia V5 Kuraray American Inc.) were directly applied between the ceramic veneer with 0.5- and 0.7-mm thicknesses and composite substrate as follow: Universal, Clear, Brown, White and Opaque. Compressive pressure (250 gr) was applied on the ceramic slices for 10 seconds using a universal testing machine and then polymerized with a light-curing unit (LITEX 680A Curing Light, Dentamerica) at 500 mw/cm^2^ for 120 seconds (each Incisal, Body, and Cervical layer for 40 seconds) (Figure 3).

### Evaluation of Color

**Figure 4:**
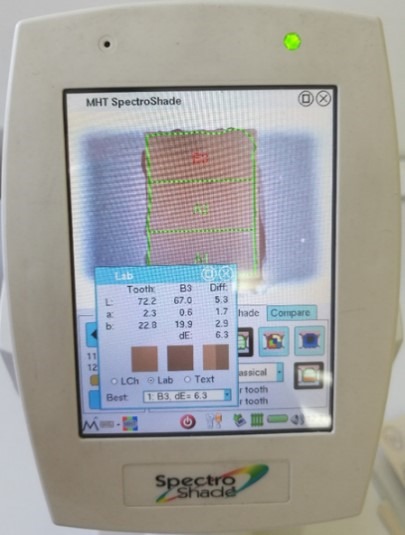
CIELab coordinate measurements by a dental spectrophotometer.

To evaluate color, the specimens were projected onto a white background.^20^ The evaluation of color parameters was determined using the CIELab system of color using a spectrophotometer (SpectroShade Micro, MHT, Verona, Italy). All SpectroShade assessments were performed by one trained operator. Before and after measurement of each slice, the instrument was evaluated in terms of calibration. The position of the device was perpendicular to the surface of the specimen and it was reproducible in order to always obtain equal measurement conditions. To evaluate shade in different areas of the specimen, the spectrophotometer in “Tooth area” mode determined the CIELab values of cervical, middle and incisal areas for each ceramic slice simultaneously. By pushing the measure button, a colored light band was emitted and the mouthpiece was precisely positioned over the specimen. The examiner could monitor the specimen via the screen; as soon as the desired position was verified and the correct geometry was indicated by a horizontal green line, the shade was recorded. While pushing the shade button, the specimen was divided into three equal zones along the median axis by Vita shades. The specimens’ L*a*b* color findings were measured after try-in paste application and cementation for three times consecutively; in order to determine the color of the specimens, the mean values were calculated. The CIE color difference was calculated for the specimens using the following formula: ΔE= [(L2-L1)2+(a2-a1)2+(b2-b1)2]1/2, in which L1, a1, b1, are ceramic values with try-in paste in place and L2, a2, b2, after cementing and curing of resin cement (Figure 4).

### Statistical Analysis

Data were analyzed with SPSS 17 (SPSS Inc., Chicago, IL, USA). ΔE differences among the groups were evaluated using Multifactor ANOVA. To determine the significant differences between the groups, post hoc Tuckey tests were used. The level of significance was established at P<0.05.

## Results

The means ± SD of ΔE values for different study groups are shown in Tables 1 and 2. The highest ΔE values were (0.5 mm: 2.57 ± 1.26) and (0.7 mm: 2.29 ±1.25) for White and Opaque shades, respectively. The lowest ΔE values for both thicknesses were related to Universal shade (0.5 mm: 0.93±0.46) and (0.7 mm: 0.85±0.29).

**Table 1:** Means ± standard deviations of ΔE values between try-in pastes and resin cements for thickness of 0.5 mm

	**Brown**	**Clear**	**Opaque**	**Universal**	**White**
**Cervical**	1.12 ± 0.29	1.37 ± 0.65	2.44 ± 1.13	0.95 ± 0.39	2.58 ± 1.49
**Middle**	1.04 ± 0.45	1.48 ± 0.51	2.12 ± 1.50	0.86 ± 0.53	2.34 ± 1.38
**Incisal**	1.40 ± 0.71	1.05 ± 0.50	1.73 ± 0.98	0.99 ± 0.48	2.81 ± 0.96
**Total**	1.19 ± 0.52	1.30 ± 0.57	2.10 ± 1.21	0.93 ± 0.46	2.57 ± 1.26

**Table 2:** Means ± standard deviations of ΔE values between try-in pastes and resin cements for thickness of 0.7 mm

	**Brown**	**Clear**	**Opaque**	**Universal**	**White**
**cervical**	1.01 ± 0.31	1.02 ± 0.45	1.99 ± 1.07	0.86 ± 0.41	1.80 ± 1.23
**middle**	0.81 ± 0.18	1.17 ± 0.53	2.15 ± 1.28	0.81 ± 0.20	1.68 ± 0.85
**incisal**	1.22 ± 0.30	1.15 ± 0.50	2.74 ± 1.41	0.87 ± 0.24	1.44 ± 0.65
**Total**	1.02 ± 0.31	1.11 ± 0.48	2.29 ± 1.25	0.85 ± 0.29	1.64 ± 0.92

Multifactor ANOVA results showed that ΔE (try-in paste‒resin cement) values were significantly affected by the resin cement shade (P<0.05) and the thickness of ceramic veneer (P<0.05). In contrast, different regions of multilayered ultra-translucent zirconia ceramic showed no significant effect on the agreement between the try-in paste and the respective resin cement (Table 3); however, the highest ΔE was related to the incisal region (1.58). Except White shade, all the ΔE values for 0.5 mm were higher than those in 0.7 mm, demonstrating a better shade agreement between the try-in paste and the respective resin cement with thicker ceramic veneers. In this study, ΔE>1 was set as the perceptible color change. The result showed that when Universal shade was applied ΔE values were <1.0 unit; therefore, it could not generate a perceptible color change in the veneers. The results of post hoc Tukey tests revealed that ΔE values for shades Clear, Universal and Brown were less than those of shades White and Opaque. Lighter shades had better shade agreement between the try-in paste and the respective resin cement.

**Table 3:** Multifactor ANOVA for the values of ΔE

**Source**	**Sum of Squares**	**df**	**Mean square**	**F**	**Sig.**
**Thickness** **Cement** **Region** Thickness * Cement	**3.818** **79.377** **1.354** **9.314**	**1** **4** **2** **4**	**3.818** **19.844** **0.677** **2.328**	**5.514** **28.656** **0.978** **3.362**	**0.020** **0.000** **0.378** **0.11**

## Discussion

Obtaining the planned color using indirect bonded restoration is a fundamental step to achieve a successful outcome in the esthetic rehabilitation treatments, consequently resulting in the satisfaction of both the dentist and patient.^8^ The try-in pastes could be used as indicators of the final color and as a guide to choose an appropriate color of the luting cement. However, controversial views about the color agreement of try-in paste and luting cement have been proposed. For color agreement evaluation different threshold settings have been presented and determination of the magnitude of difference between the color shade of two specimens, while it is perceptible or acceptable, is of great clinical importance.^11^ The perceptible ΔE threshold in different studies ranged from 1.0 to 3.7^21-23^ and the acceptable ΔE threshold ranged from 1.7 to 6.8.,^25^ Due to different threshold settings by the authors, different conclusions might be drawn. Nevertheless, the most widely accepted values for ΔE in the literature are 1.0 and 3.3 units for perceptibility and acceptability of color change, respectively.,^26^ In the present study, the color differences are considered visually perceptible and clinically acceptable for ΔE>1 and ΔE<3.3, respectively.

Since most shades produced perceptible color change (ΔE>1.0), the first null hypothesis was rejected. The results indicated that the color matching of try-in paste and corresponding resin cement is not always achieved. This finding accords with other studies which demonstrated that in most cases, the color of the try-in paste did not correspond to the color of its respective resin cement.^10,27,28^ In contrast, Xing et al^11^ reported high agreement between try-in pastes and their respective resin cements; however, the perceptible threshold of ΔE was considered 2 and the used body materials only represent the middle one-third of veneer restoration whereas final color is the outcome of cervical, body and incisal materials together.

The ΔE values of White and Opaque shades were highly above the perceptible limit and considered clinically significant. The opaque shade obviously increases the brightness and L value and decreases chroma. The clinical purpose of using an opaque shade is frequently to mask the undesired color of underlying substrate.^29^

The results revealed that the ceramic thickness had a significant effect on the agreement of try-in paste and corresponding resin cement (P<0.05); therefore, the second null hypothesis was rejected. The mean ΔE values at 0.5-mm thickness were significantly higher than that of 0.7-mm thickness in different shade groups; the results were in agreement with those of the previous studies that showed as the thickness of ceramic veneers reduced, higher ΔE values were achieved between try-in pastes and resin cements.^8,15^ This can be explained by the fact that as the thickness of the specimens reduces, the translucency parameters increase greatly. Therefore, greater translucency reduces the impact of the microstructure and composition of the ceramic materials on color differences between try-in paste and the respective cement shade. Also Piers et al showed that increasing the ceramic thickness increases its opacity and hinders transmission of light, which favors light scattering and reduces translucency.^30^ In contrast, Xing et al^11^ reported that the thickness did not affect the agreement of try-in pastes and the respective resin cements. This might be due to the use of Ceromer disks which have microstructure and composition different from cubic zirconia used in the present study. The cubic zirconia used in this study is ultra-translucent multilayered zirconia (UTML) that is the third generation of dental zirconia with 5% mol yttria. The cubic zirconia in different crystallographic directions has isotropic state which decreases the light scattering occurring at grain boundaries.^31^ Accordingly, the cubic zirconia has higher translucency properties. Color and other optical features such as scattering and absorption of light are important for shade matching and esthetic outcomes. Furthermore, this multilayered zirconia was polychromatic to resemble the optical properties similar to natural teeth following insertion of dyes in framework blocks within the gradient chromatic ceramic disks.

The results of this study showed that different regions of multilayered ceramic veneers have no significant effect on the agreement of try-in pastes and resin cement shades (P>0.05); therefore, the third hypothesis was confirmed. However, the highest ΔE was related to incisal regions (1.58); this might be due to the presence of lower dye components in the incisal layer and different degrees of translucency across the layers.^32^ Ueda et al revealed that different layers of UTML disc have different transmittance values.^33^

In spite of the fact that there was a perceptible color difference between the resin cement and corresponding try-in paste, since most of the ΔE values were higher than the perceptible threshold (ΔE>1), all the ΔE values derived from the specimens were below the clinically unacceptable threshold of ΔE<3.3.

Using only one shade of substrate (A3) can be considered as a limitation of the current study. In addition, only shade A1 of ultra-translucent multilayered zirconia veneer was applied in the study. Further studies on other shades of substrate and zirconia veneer are recommended.

## Conclusion

The following conclusions can be drawn within the limitations of this in vitro study:

1. Perceptible color differences were found between the try-in pastes and the respective resin cement in most shades investigated.
2. The thickness of the ultra-translucent multilayered zirconia veneer affected the agreement of the try-in pastes and the respective resin cement.
3. The regions of multilayered zirconia veneer did not affect the agreement of the try-in pastes and the respective resin cement.

## Authors’ contributions

The study was planned by MD, FP, SK. The literature review was performed by MB, FP, MD and SK. PA and SA performed the experiments and drafted the manuscript. SK, MD and FP performed the experimental procedure. The statistical analyses and interpretation of data were carried out by FP. All the authors critically revised the manuscript for intellectual content. All the authors have read and approved the final manuscript.

## Acknowledgments

The authors thank all our friends and members of staff of our departments, who encouraged us to complete the study.

## Funding

The study was sponsored by Dental and Periodontal Research Center, Tabriz University of Medical Sciences, Tabriz, Iran.

## Competing interests

The authors declare no competing interests with regards to the authorship and/or publication of this article.

## Ethics approval

The study was supported by Dental Faculty of Dentistry, Tabriz University of Medical Sciences. The study protocol was approved by the Ethics Committee at Tabriz University of Medical Sciences (IR.TBZMED.REC.1396.844).

## References

[1] Rafael CFDPL, Maria Munõz, Meredith Tercero; D'Altoé Garbelotto, Luis Gustavo; Liebermann, Anja; Özcan, Mutlu; Maziero Volpato, Cláudia Ângela (2017). Optical Factors: Affecting Anterior Esthetics in All-Ceramic Restorations: Two Case Reports. Journal of Cosmetic Dentistry.

[2] Jankar AS, Kale Y, Pustake S, Bijjaragi S, Pustake B (2015). Spectrophotometric study of the effect of luting agents on the resultant shade of ceramic veneers: an invitro study. J Clin Diagn Res.

[3] Rondoni D (2016). Zirconia: Some practical aspects from the technologist’s point of view. Int J Esthet Dent.

[4] Souza R, Barbosa F, Araújo G, Miyashita E, Bottino M, Melo R (2018). Ultrathin Monolithic Zirconia Veneers: Reality or Future? Report of a Clinical Case and One-year Follow-up. Oper Dent.

[5] Al‐Amleh B, Lyons K, Swain M (2010). Clinical trials in zirconia: a systematic review. J Oral Rehabil.

[6] McLaren EA, Lawson N, Choi J, Kang J, Trujillo C (2017). New High-Translucent Cubic-Phase–Containing Zirconia: Clinical and Laboratory Considerations and the Effect of Air Abrasion on Strength. Compend Contin Educ Dent.

[7] Turgut S, Bagis B, Ayaz EA (2014). Achieving the desired colour in discoloured teeth, using leucite-based cad-cam laminate Systems. J Dent.

[8] Vaz EC, Vaz MM, Torres ÉM, Souza JB, Barata TdJE, Lopes LG. Resin Cement: Correspondence with Try‐In Paste and Influence on the Immediate Final Color of Veneers. J Prosthodont. 2018 (in press). 10.1111/jopr.12728 29314449

[9] Uludag B, Ozturk O, Ozturk AN (2009). Microleakage of ceramic inlays luted with different resin cements and dentin adhesives. J Prosthet Dent.

[10] Balderamos LP, O'Keefe KL, Powers JM (1997). Color accuracy of resin cements and try-in pastes. Int J Prosthodont.

[11] Xing W, Jiang T, Ma X, Liang S, Wang Z, Sa Y (2010). Evaluation of the esthetic effect of resin cements and try-in pastes on ceromer veneers. J Dent.

[12] Vaz E, Vaz M, de Oliveira Rodrigues MG, Takano A, Gonzaga LL (2016). Try-in Pastes Versus Resin Cements: A Color Comparison. Compend Contin Educ Dent.

[13] Rigoni P, Amaral FLBd, França FMG, Basting RT (2012). Color agreement between nanofluorapatite ceramic discs associated with try-in pastes and with resin cements. Braz Oral Res.

[14] Xu B, Chen X, Li R, Wang Y, Li Q (2014). Agreement of Try‐In Pastes and the Corresponding Luting Composites on the Final Color of Ceramic Veneers. J Prosthodont.

[15] Mourouzis P, Koulaouzidou E, Palaghias G, Helvatjoglu-Antoniades M (2018). Color match of luting composites and try-in pastes: the impact on the final color of CAD/CAM lithium disilicate restorations. Int J Esthet Dent.

[16] ALGhazali N, Laukner J, Burnside G, Jarad F, Smith P, Preston A (2010). An investigation into the effect of try-in pastes, uncured and cured resin cements on the overall color of ceramic veneer restorations: an in vitro study. J Dent.

[17] Alghazzawi TF, Lemons J, Liu P-R, Essig ME, Janowski GM (2012). The failure load of CAD/CAM generated zirconia and glass-ceramic laminate veneers with different preparation designs. J Prosthet Dent.

[18] AlQAHTANI MQ, AlJURAIS RM, AlSHAAFI MM (2012). The effects of different shades of resin luting cement on the color of ceramic veneers. Dent Mater J.

[19] Çömlekoğlu ME, Paken G, Tan F, Dündar‐Çömlekoğlu M, Özcan M, Akan E (2016). Evaluation of different thickness, die color, and resin cement shade for veneers of multilayered CAD/CAM blocks. J Prosthodont.

[20] Zenthöfer A, Cabrera T, Corcodel N, Rammelsberg P, Hassel AJ (2014). Comparison of the Easyshade Compact and Advance in vitro and in vivo. Clin Oral Investig.

[21] Seghi RR, Hewlett E, Kim J (1989). Visual and instrumental colorimetric assessments of small color differences on translucent dental porcelain. J Dent Res.

[22] Douglas RD, Steinhauer TJ, Wee AG (2007). Intraoral determination of the tolerance of dentists for perceptibility and acceptability of shade mismatch. J Prosthet Dent.

[23] Kuehni RG, Marcus RT (1979). An experiment in visual scaling of small color differences. Color Research & Application.

[24] Douglas RD, Brewer JD (1998). Acceptability of shade differences in metal ceramic crowns. J Prosthet Dent.

[25] Ruyter I, Nilner K, Möller B (1987). Color stability of dental composite resin materials for crown and bridge veneers. Dent Mater.

[26] Lindsey DT, Wee AG (2007). Perceptibility and acceptability of CIELAB color differences in computer-simulated teeth. J Dent.

[27] Wang X, Powers J (1999). Color differences between a resin cement and try-in paste. Zhonghua Kou Qiang Yi Xue Za Zhi.

[28] Kampouropoulos D, Gaintantzopoulou M, Papazoglou E, Kakaboura A (2014). Colour matching of composite resin cements with their corresponding try-in pastes. Eur J Prosthodont Restor Dent.

[29] Dede DÖ, Sahin O, Özdemir OS, Yilmaz B, Celik E, Köroğlu A (2017). Influence of the color of composite resin foundation and luting cement on the final color of lithium disilicate ceramic systems. Journal of Prosthetic Dentistry.

[30] Pires LA, Novais PM, Araújo VD, Pegoraro LF (2017). Effects of the type and thickness of ceramic, substrate, and cement on the optical color of a lithium disilicate ceramic. Journal of Prosthetic Dentistry.

[31] Kwon SJ, Lawson NC, McLaren EE, Nejat AH, Burgess JO. Comparison of the mechanical properties of translucent zirconia and lithium disilicate. J Prosthet Dent. 2018 (in press). 10.1016/j.prosdent.2017.08.004 29310875

[32] Shamseddine L, Majzoub Z (2017). Relative Translucency of a Multilayered Ultratranslucent Zirconia Material. J Contemp Dent Pract.

[33] Ueda K, Gueth J-F, Erdelt K, Stimmelmayr M, Kappert H, Beuer F (2015). Light transmittance by a multi-coloured zirconia material. Dent Mater J.

